# CRISPR/Cas9-based knockout of *BnaLYK* compromises pattern-triggered immunity and resistance to *Sclerotinia sclerotiorum* in *Brassica napus*

**DOI:** 10.1186/s12870-025-07823-w

**Published:** 2026-04-13

**Authors:** Yingjun Liu, Xiaowen Yin, Pinyuan Jin, Huajian Zhang

**Affiliations:** 1 Key Laboratory of Agri-products Quality and Biosafety (Ministry of Education), Anhui Agricultural University, Hefei, 230036 China; 2 Anhui Province Key Laboratory of Crop Integrated Pest Management, Department of Plant Pathology, College of Plant Protection, Anhui Agricultural University, Hefei, 230036 China

**Keywords:** Brassica napus, Sclerotinia stem rot, LYK1, Plant immunity, CRISPR/Cas9

## Abstract

Rapeseed (*Brassica napus*) is severely threatened by *Sclerotinia sclerotiorum*, the causal agent of Sclerotinia stem rot (SSR). The lack of fully resistant cultivars, combined with the genetic complexity of rapeseed as an allopolyploid species, has hindered the identification of major resistance genes for molecular breeding strategies. This study identifies the involvement of BnaLYK, a LysM receptor-like kinase, in disease resistance and immune signaling pathways. The two homologs, BnaA05.LYK and BnaC05.LYK, function as orthologs of AtLYK1 in *B. napus*. Both genes were induced upon *S. sclerotiorum* infection and were localized to the plasma membrane. Heterologous expression of either gene in the *atlyk1* mutant restored chitin-triggered reactive oxygen species (ROS) burst, callose deposition, and defense gene expression. CRISPR/Cas9-mediated knockout of *BnaLYK* significantly compromised resistance against *S. sclerotiorum*, characterized by increased necrotic lesion formation and decreased expression levels of defense-related genes. BnaLYK was shown to be indispensable for chitin- and peptidoglycan-induced pattern-triggered immunity, regulating ROS production, callose accumulation, and defense gene expression. Our findings demonstrate BnaLYK is essential for chitin-induced immunity and basal resistance against *S. sclerotiorum* in rapeseed, which underscore the conserved functionality of LysM receptors in polyploid crops and offer a promising target for breeding SSR-resistant rapeseed varieties.

**Supplementary Information:**

The online version contains supplementary material available at 10.1186/s12870-025-07823-w.

## Introduction

Rapeseed (*Brassica napus*) is a globally important oilseed crop that serves as a primary source of edible vegetable oil and biodiesel feedstock. Beyond its economic value in agriculture and energy sector, rapeseed also has diverse applications in tourism, chemical industry, and livestock nutrition. However, the increasing cultivation of rapeseed has been accompanied by increasing threats from pathogenic diseases. Among these, Sclerotinia stem rot (SSR), caused by necrotrophic fungus *Sclerotinia sclerotiorum*, represents the most devastating disease affecting rapeseed production worldwide [1, 2]. SSR also compromises the quality of rapeseed oil and even endangers human and animal health through mycotoxin contamination [3]. The challenge in combating SSR has multiple biological complexities. As an allopolyploid (AACC) derived from the natural hybridization of *Brassica rapa* (AA) and *Brassica oleracea* (CC), rapeseed exhibits intricate patterns of disease resistance traits [4]. The sophisticated pathogenicity mechanisms of *S. sclerotiorum*, coupled with the limited physiological differentiation among pathogen strains, have hindered the development of reliable pathosystems for resistance gene mapping [5, 6]. Despite extensive screening of rapeseed germplasm, no cultivars exhibiting complete resistance to *S. sclerotiorum* have been identified to date [3, 7, 8]. These constraints have severely impeded progress in cloning of major resistance genes and developing molecular breeding strategies for SSR management.

Plants defend themselves against pathogens using a sophisticated innate immune system shaped by long-term co-evolution with host-pathogen. The primary line of defense, known as PAMP-Triggered Immunity (PTI), is initiated when pattern recognition receptors (PRRs) recognize pathogen-associated molecular patterns (PAMPs). This conserved defense mechanism was first characterized in the seminal work of Jones and Dangl [9]. Various PAMPs, including bacterial lipopolysaccharide (LPS), peptidoglycan (PGN), and fungal chitin oligomers, have been shown to elicit robust immune activities across both animals and plants kingdoms [10]. This recognition process depends on specific interaction between PAMPs and their cognate PRRs, which subsequently activate phosphorylation cascades that modulate downstream defense regulators, culminating in comprehensive antimicrobial responses [11–13]. Effective PTI activation enables plants to establish basal resistance that can restrict pathogen colonization and growth.

Among the key PRRs involved in fungal recognition are the LysM domain-containing receptor-like kinases (LYK), which specialize in perceiving signaling from fungal pathogens [14]. The LysM domain is an evolutionarily conserved protein module of approximately 40 amino acids, initially identified in bacterial secretory hydrolases [15]. Structurally, this domain features a distinctive architecture comprising antiparallel β-sheet flanked by α-helices, enabling specific carbohydrate binding [16, 17]. Comparative genomic analyses reveal variable expansion of LYK gene families across plant species: *Arabidopsis thaliana* possesses five LYK members, while rice (*Oryza sativa*) contains ten homologs [18–20]. Functional studies in model plants have elucidated crucial roles for LYKs in microbial perception and immune activation. In rice, the chitin-binding protein CEBiP was the first PRR shown to be essential for chitin signaling, while OsCERK1 was subsequently demonstrated to physically interact with CEBiP to coordinate chitin-triggered defenses [21–23]. Similarly, Arabidopsis employs multiple LYKs (including CERK1/LYK1, LYK4, and LYK5) as chitin receptors, whereas LYK3 appears to function as a negative immune regulator [24–27]. Structural studies have revealed that receptor dimerization (of either CERK1 or CEBiP) constitutes a critical step in immune signal transduction [28, 29]. Despite these advances in model systems, the functional repertoire of LYKs in rapeseed-pathogen interactions remains largely unexplored.

This study elucidates the immunological functions of BnaLYK in rapeseed’s defense against SSR. We demonstrate that BnaLYK modulates resistance to *S. sclerotiorum* by orchestrating chitin-induced immune outputs, including reactive oxygen species (ROS) burst, callose deposition, and transcriptional activation of defense-related genes. Through molecular genetic approaches, we establish BnaLYK’s role in PTI signaling and its contribution to SSR resistance. Our findings provide novel insights into the mechanistic basis of rapeseed immunity against fungal pathogens, offering both theoretical frameworks for plant-microbe interactions and practical targets for molecular breeding of disease-resistant rapeseed cultivars.

## Methods

### Plant materials

*B. napus* Westar was used for gene knockout test based on the CRISPR/Cas9 gene editing technique. *B. napus* were grown in a growth chamber at 22℃ with a 16 h photoperiod. All *Arabidopsis thaliana* lines used were in the Columbia (Col-0) background. The T-DNA insertion line *Atlyk1* (At3g21630) (SALK_032940) was obtained from the Arabidopsis Biological Resource Centre (ABRC, http://abrc.osu.edu/). *A. thaliana* was grown in growth chambers under long-day conditions (16-h light/8-h dark) at 21℃, with a light intensity of 80 to 100 µE m^− 2^ s^− 1^.

### Bioinformatic analysis

The sequences of the *BnaLYK* homologous gene were blasted against the National Center for Biotechnology Information (NCBI) database to search for similar sequences from different species. Both gene and amino acid sequences of the results were downloaded. The amino acid sequences were used to construct phylogenetic trees using MEGA (https://www.megasoftware.net) software and analyzed using DNAMAN (https://www.lynnon.com/). Finally, online analysis tools from NCBI (https://www.ncbi.nlm.nih.gov/guide/domains-structures/) and SMART (https://smartembl.de/) were used to predict the conserved structures of the genes.

### Subcellular localization of *BnaLYK*

The full-length CDS sequence of *BnaLYK* was obtained without specific deletion of the stop codon. The two homologous full-length CDS sequences were constructed separately into the pH7LIC6.0 vector and fused with GFP to obtain the fusion protein vectors 35S:*BnaA05.LYK1*-GFP, 35S:*BnaC05.LYK1*-GFP. After transforming Agrobacterium, subcellular localization of the proteins was performed by injecting the bacteria into the lower epidermal cells of tobacco leaves for transient expression. Two days later, confocal microscopy was used to observe fluorescent signals.

### CRISPR/Cas9-mediated mutations of *BnaLYK*

The CRISPR-P 2.0 online target design tool (http://crispr.hzau.edu.cn/CRISPR2/) was used to design target sites for *BnaLYK*. PKSE401 was used as the vector backbone to construct the gene editing vector, which was transformed into Westar via Agrobacterium-mediated genetic transformation. After PCR identification and detection of editing events, homozygous transgenic-positive plants were obtained in the T2 generation. The sequence of target 1 was CCGTGGAATCTAAATGCAGTGG, and the sequence of target 2 was TTACCGCAAAAGCAGTTCACGG.

### Assessment of *B. napus* resistance to *S. sclerotiorum*

The *S. sclerotiorum* was cultured on potato dextrose agar (PDA, Becton, Dickinson and Company, USA) at 23 °C in the dark. Rapeseed leaves were placed in Petri dishes lined with cotton and filter paper to maintain humidity. Mycelial plugs taken from the actively growing margins were used to inoculate the upper to middle portions of the leaves, avoiding the main veins. After inoculation, the leaves were uniformly misted with sterile distilled water. Disease symptoms were monitored and photographed at 0, 24, 36, and 48 h post-inoculation (hpi), and lesion areas were measured accordingly.

### Gene expression analysis

An RNAprep Pure Plant Kit (Tiangen Biotech Co. Ltd., Beijing, China) was used to extract total RNA from plant samples. A HiScript^®^ III First Strand cDNA Synthesis Kit (gDNA Wiper) (Vazyme Biotech Co. Ltd., Nanjing, China) was used to synthesize cDNA from total RNA (1 µg) following the manufacturer’s instructions, and qRT-PCR was performed using developed specific primers (Table S1). The qRT-PCR system and program referenced the ChamQ Universal SYBR^®^ qPCR Master Mix (Vazyme Biotech Co. Ltd., Nanjing, China) and the Bio-Rad CFX384TM real-time detection system (Bio-Rad, Hercules, CA, USA). Each gene’s qRT-PCR included three biological replicates and three technical replicates.

### Callose deposition detection

Leaves were cleared and incubating with 95% ethanol until they attained a transparent state. Next, they were stained with 0.01% aniline blue dissolved in 0.07 M phosphate buffer (pH = 8) for 2 h. Subsequently, callose deposits were visually examined under ultraviolet illumination by using a microscope.

### Reactive oxygen species detection

ROS production was quantified as detailed in a previous study by D’Ambrosio et al. [30]. Leaf disks from Arabidopsis or rapeseed were floated overnight in deionized water. To induce ROS production, 100 µg/mL of chitin or peptidoglycan was applied simultaneously with 20 mM luminol (Sigma-Aldrich) and 0.02 mg/mL horseradish peroxidase (Sigma-Aldrich). Luminescence was recorded using a luminometer (Berthold Technologies) for a duration of 1 h after processing.

### In vitro phosphorylation assay

Phosphorylation assays were performed according to the method described by Zhang et al. [31]. For each treatment, 100 mg of leaves were treatment with chitin, peptidoglycan or ddH₂O for 15 min. The sources and dilutions of the antibodies were as specified: α-P44/42 antibody (1:10,000; Cell Signaling Technology, Beverly, MA, USA) and goat anti-mouse horseradish peroxidase-conjugated antibody (1:5,000; Abmart, Shanghai, China).

## Results

### *BnaA05.LYK* and *BnaC05.LYK* encode two LYKs localized in the plasma membrane

**Fig. 1:**
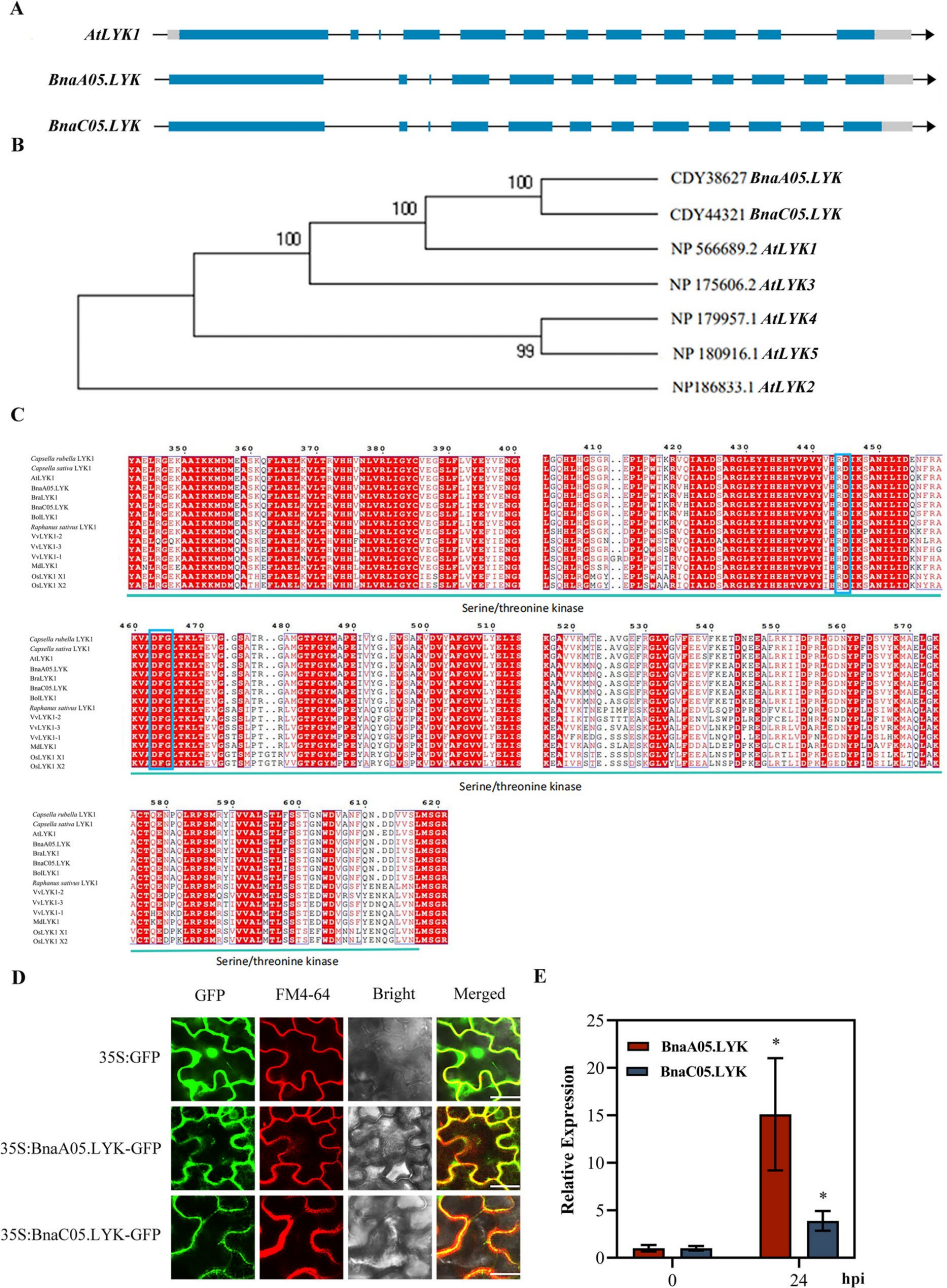
Bioinformatics analysis of *BnaLYK.* (**A**) DNA sequence analysis of *BnaLYK* with *AtLYK1.* (**B**) Analysis of the evolutionary tree of BnaLYKs with the Arabidopsis LYK family. (**C**) Analysis of protein structural domains of LYK1 family members. RD and DFG sequences were shown by blue boxes. (**D**) Subcellular localization of BnaLYKs. The structures obtained by fusing the coding sequences of BnaA05.LYK and BnaC05.LYK with GFP tags were expressed in tobacco (Nicotiana benthamiana) epidermal cells, respectively. FM4-64 images show membrane staining. Images were taken by laser confocal microscopy. Bars = 25 μm. (**E**) The expression of BnaLYK induced by Sclerotinia sclerotiorum. Asterisks indicate statistical significance from control group, determined via Student’s *t*-test (*: *P* <0.05)

To investigate the molecular mechanisms underlying resistance to *S. sclerotiorum* in rapeseed, we inoculated rapeseed plants with *Sclerotinia sclerotiorum* and performed transcriptome sequencing. A suspension of *S. sclerotiorum* was sprayed onto *B. napus *leaves, and the inoculated plants were continuously observed for 10 h. Lesion areas were detectable under a microscope by 10 h post inoculation (hpi) and leaf wrinkling occurred. Subsequent RNA-sequencing showed that two of the differentially expressed genes were annotated as members of the LYK family. This search identified two homologous genes in *Brassica napus*: one located on chromosome A05 (designated *BnaA05.LYK*) and the other on chromosome C05 (designated *BnaC05.LYK*). The full-length coding sequences of both genes were amplified via PCR from *B. napus*. Sequence analysis showed that *BnaA05.LYK* and *BnaC05.LYK* exhibited complete identity with their respective entries and possessed the same exon-intron structures as *AtLYK1* from *Arabidopsis* (Fig. 1A). Phylogenetic analysis was conducted using protein sequences of the two *BnaLYK* genes and other members of the *Arabidopsis thaliana* LYK family (Fig. 1B and C). The resulting phylogenetic tree demonstrated that *BnaA05.LYK* and *BnaC05.LYK* clustered closely with *AtLYK1*, confirming their orthologous relationship. Structural analysis of the encoded proteins revealed that both BnaA05.LYK and BnaC05.LYK contained a signal peptide, three extracellular LysM domains, a transmembrane domain, and a cytoplasmic serine/threonine kinase domain. The RD and DFG motifs, which are critical for kinase activity, were highly conserved in BnaLYKs compared to LYK homologs from other plant species. Subcellular localization was examined using GFP fusion constructs (35 S:*BnaA05.LYK*-GFP and 35 S:*BnaC05.LYK*-GFP), which confirmed plasma membrane localization. Fluorescence microscopy showed that green fluorescent signals colocalized with the plasma membrane marker FM4-64, consistent with their predicted receptor kinase localization (Fig. 1E). This finding suggests BnaLYKs and AtLYK1 are functional orthologs and may play similar roles in mediating plant immune responses in *B. napus*.

### BnaLYKs restore the immune response of *Arabidopsis* to chitin

**Fig. 2:**
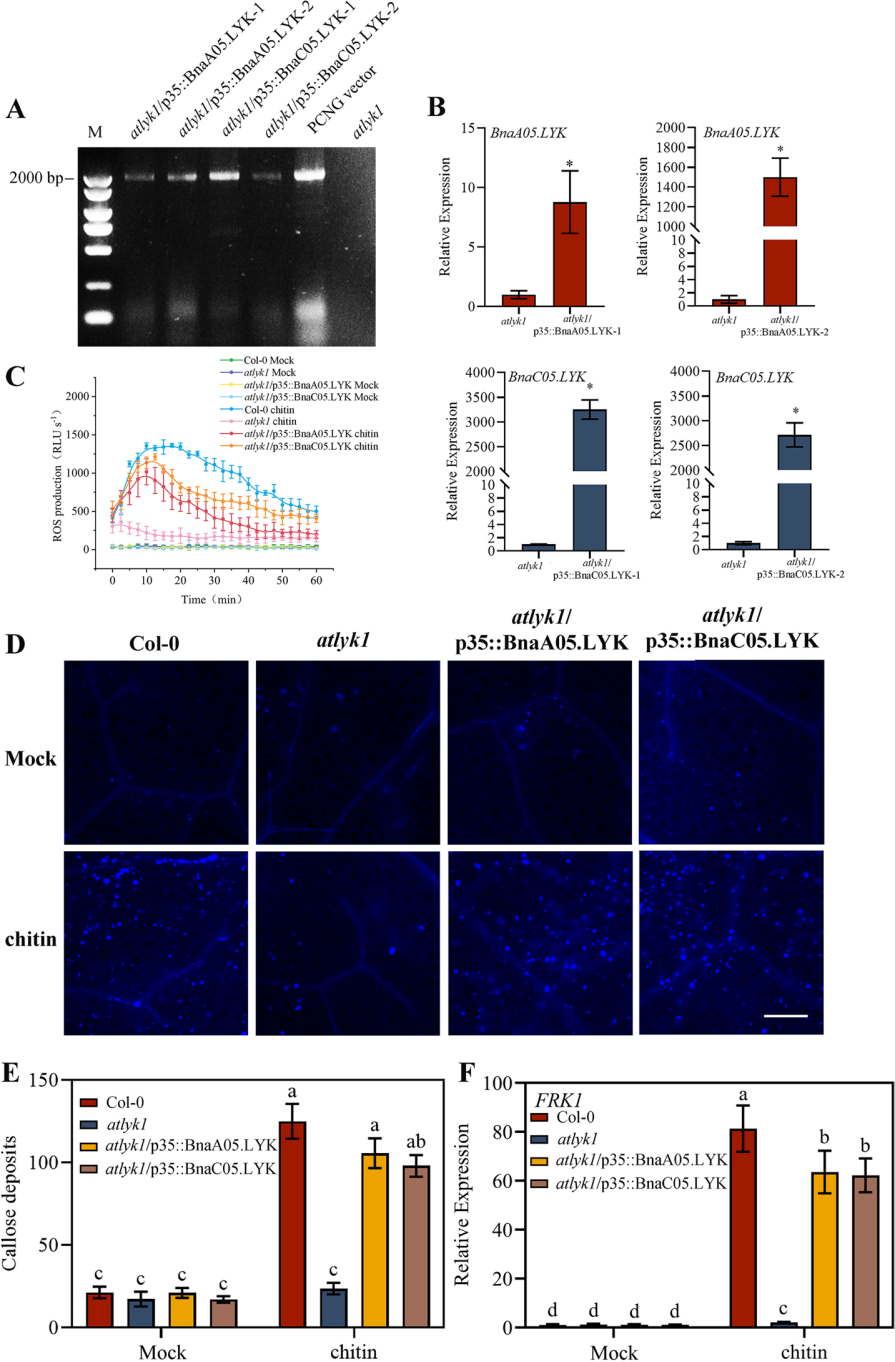
*BnaLYK* can restore the immune response of *atlyk1* mutants to chitin. (**A**) Screening for *atlyk1*/p35S::BnaLYK plants. (**B**) The expression levels of *BnaA05.LYK* and *BnaC05.LYK* in transgenic lines. Asterisks indicate statistical significance, determined via Student’s *t*-test (*: *P* < 0.05). (**C**) Chitin-induced cellular ROS burst was determined and expressed in relative light units (RLU) (**D**, **E**) Callose deposition induced by chitin. (**F**) Relative expression of a defense gene encoding *flagellin-induced receptor kinase1* (*FRK1*). Student’s *t*-test: different letters indicate significant differences, *P* < 0.05. Bars = 25 μm

The phylogenetic analysis revealed that both *BnaA05.LYK* and *BnaC05.LYK* cluster in close proximity to *AtLYK1*, confirming their orthologous relationship. To investigate whether these genes are involved in plant defense responses against pathogens, *Brassica napus* cv. Westar plants were inoculated with *S. sclerotiorum*. Quantitative RT-PCR analysis showed significant upregulation of *BnaA05.LYK* and *BnaC05.LYK* transcripts following fungal infection, indicating their induction by *S. sclerotiorum* (Fig. 1D). Transgenic *Arabidopsis Atlyk1* mutants expressing either *BnaA05.LYK* or *BnaC05.LYK* were generated to determine whether *BnaA05.LYK* and *BnaC05.LYK* can restore immunity (Fig. 2A and B). Previous studies have demonstrated that *Atlyk1* mutants exhibit impaired chitin responsiveness due to their inability to mount immune responses to this fungal elicitor. In wild-type Col*-*0 plants, chitin treatment induced a robust reactive oxygen species (ROS) burst, while *Atlyk1* mutants displayed negligible ROS production. Transgenic lines *Atlyk1/p35S::BnaA05.LYK-1* and *Atlyk1/p35S::BnaC05.LYK-2* restored ROS levels to those of wild-type Col-0 plants, indicating functional complementation of the chitin response defect. Similarly, chitin-induced callose deposition was abolished in *Atlyk1* mutants but fully restored in both transgenic lines (Fig. 2C, D and E). We also monitored the expression of *FLG22-INDUCED RECEPTOR-LIKE KINASE1* (*FRK1*), a chitin-responsive defense gene encoding flagellin-induced receptor kinase 1. Chitin treatment induced an ~ 81-fold increase in *FRK1* transcription in Col*-*0 plants relative to mock-treated controls, whereas *Atlyk1* mutants exhibited a severe ~ 40-fold reduction (Fig. 2F). Transgenic *Atlyk1* lines expressing *BnaLYK* showed a ~ 30-fold upregulation of *FRK1* compared to the *Atlyk* lines, demonstrating partial restoration of chitin signaling. Collectively, these results confirm that *BnaA05.LYK* and *BnaC05.LYK* functionally complement *Atlyk1* mutant defects in chitin-mediated ROS production, callose deposition, and *FRK1* activation.

### Knockout of *BnaLYK *compromises the resistance of rapeseed to *S. sclerotiorum*

**Fig. 3:**
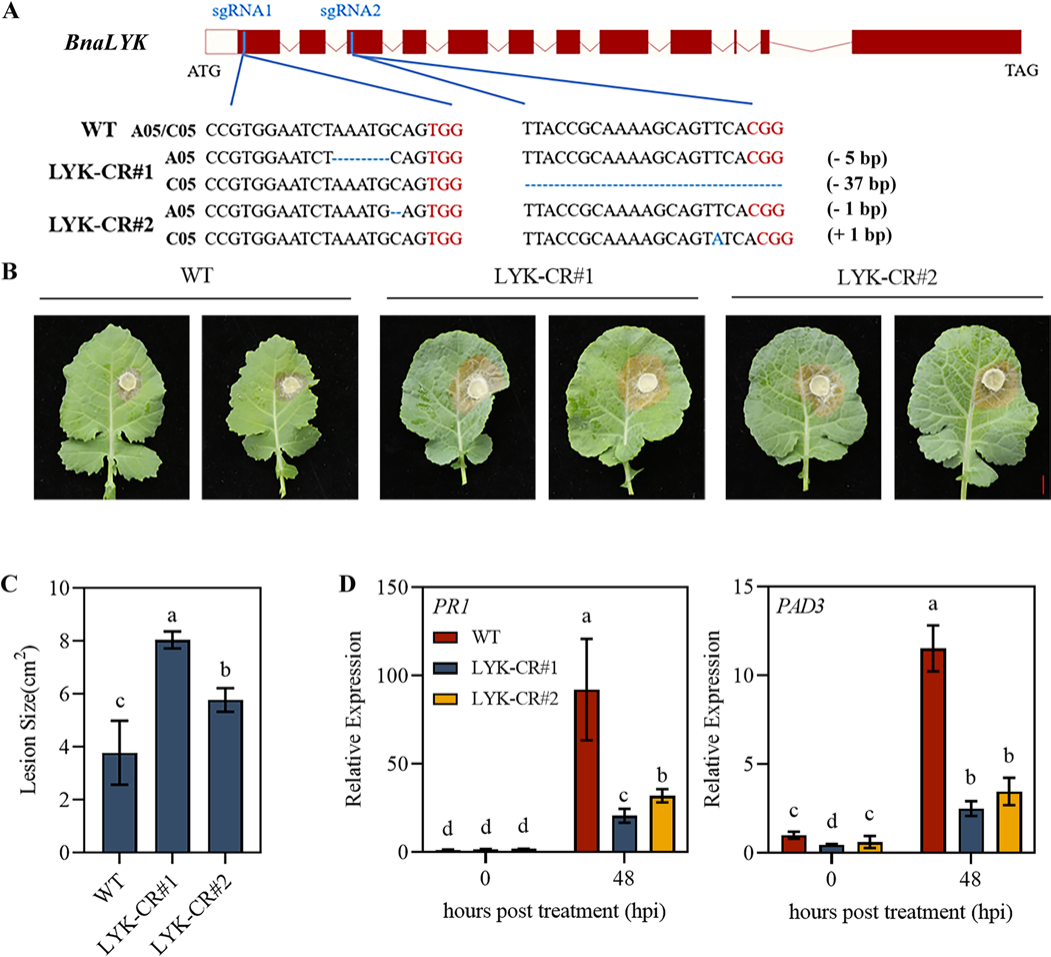
The bnalyk mutants show reduced resistance to *S. sclerotiorum.* (**A**) Schematic sequence of *BnaLYK* gene editing target and editing form detection. (**B**) Phenotypes of WT (Westar), *bnalyk* mutations (LYK-CR#1, and LYK-CR#2) rapeseed lines after infection with *S. sclerotiorum* at 48 hpi, Bar = 1 cm. (**C**) The lesion size in rapeseed infected with *S. sclerotiorum *at 48 hpi. (**D**) Expression levels of defense-related gene *PR1* and *PAD3* in rapeseed infected with *S. sclerotiorum* at 48 hpi. Student’s *t*-test: different letters indicate the values are significantly different, *P* < 0.05

We next constructed CRISPR/Cas9-mediated genetic transformation constructs targeting *BnaA05.LYK* and *BnaC05.LYK* to elucidate the biological functions of *BnaLYK*. Two suitable target sites were identified. The first target site, sgRNA1, was located in the first exon of both *BnaA05.LYK* and *BnaC05.LYK*, while the second target site, sgRNA2, was located in their third exon (Fig. 3A). Two independent mutant lines were identified following transgenic screening and sequencing validation, exhibiting distinct mutation types (deletions and insertions) within the *BnaLYK* gene locus. These lines were self-pollinated to the T2 generation, resulting in the successful isolation of pure homozygous *BnaLYK* gene-edited plants. Pathogen inoculation assays were conducted using the *bnalyk* mutant lines and the wild-type cultivar Westar (WT) as a control to test its potential defense-related functions. Four-week-old plants were inoculated with *S. sclerotiorum* on detached leaves, and disease symptoms were recorded 48 hpi. Compared to WT plants, both mutant lines developed significantly larger necrotic lesions, indicating compromised resistance to *S. sclerotiorum* infection (Fig. 3B and C). *PR1* (*pathogenesis-related protein 1*) and *PAD3* (*PHYTOALEXIN DEFICIENT 3*) are two defense-related genes, which are known to contribute immune response against *S. sclerotiorum*. *PR1* and *PAD3* transcript levels in *bnalyk* mutants were significantly downregulated compared to WT plants following pathogen infection (Fig. 3D). These results collectively demonstrate that *BnaLYK* mutation compromises *B. napus* resistance to *S. sclerotiorum*, along with suppression of key defense-related genes.

### Knockout of *BnaLYK *impairs the induced resistance of rapeseed to *S. sclerotiorum*

**Fig. 4:**
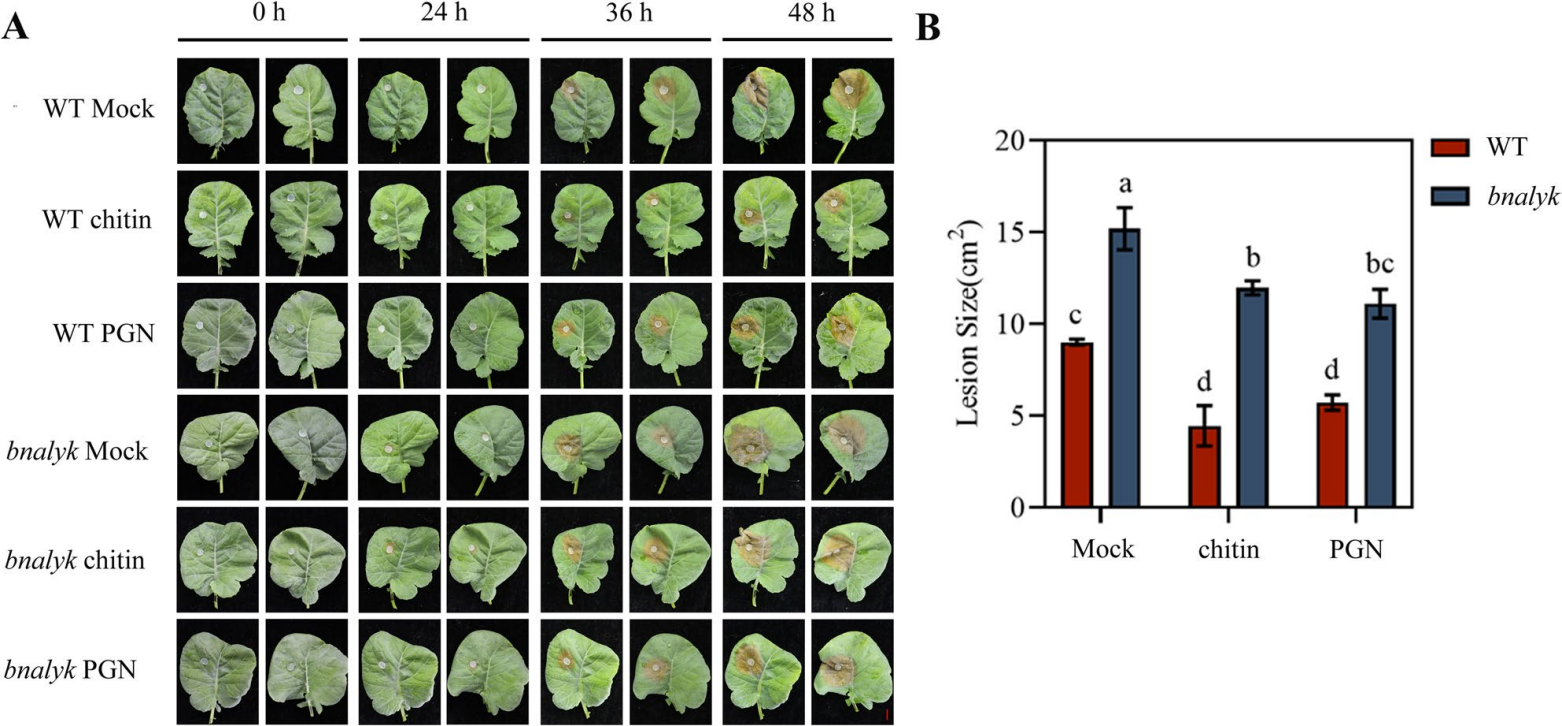
The *bnalyk* mutants treated with chitin or PGN show reduced resistance to *S. sclerotiorum*. WT and *bnalyk* lines were pre-treated with H_2_O (Mock), chitin and PGN for 24 h, and subsequently inoculated with *S. sclerotiorum*. (**A**) Phenotype of rapeseed after 24, 36 and 48 h inoculation with *S. sclerotiorum*, Bar = 1 cm. (**B**) The lesion size in rapeseed inoculation with *S. sclerotiorum* after 48 h

BnaLYK function in PAMP-triggered immunity against fungal infection was further explored. To this end, symptom severity differences were evaluated between WT and *bnalyk* mutant plants following chitin pretreatment prior to *S. sclerotiorum* infection. Both WT and mutant plants were pretreated with peptidoglycan (PGN, a bacterial PAMP) or chitin (a fungal PAMP) to evaluate their effects on resistance against *S. sclerotiorum*. The results showed that there were distinct differences in the disease resistance responses among different genotypes. Under chitin pretreatment, WT plants exhibited significantly reduced lesion sizes and enhanced resistance to *S. sclerotiorum* compared to non-pretreated controls (Fig. 4A and B). In contrast, *bnalyk* mutant plants showed no improvement in resistance following chitin pretreatment, with lesion sizes comparable to non-pretreated controls. Similarly, PGN pretreatment induced significant resistance in WT plants, reduced lesion size by ~ 50%, but had no effect on mutant plants. These results indicate that *BnaLYK* is essential for PAMP-triggered resistance against *S. sclerotiorum* in *B. napus*.

### Knockout of *BnaLYK* affects the immune response of rapeseed to chitin and PGN

**Fig. 5:**
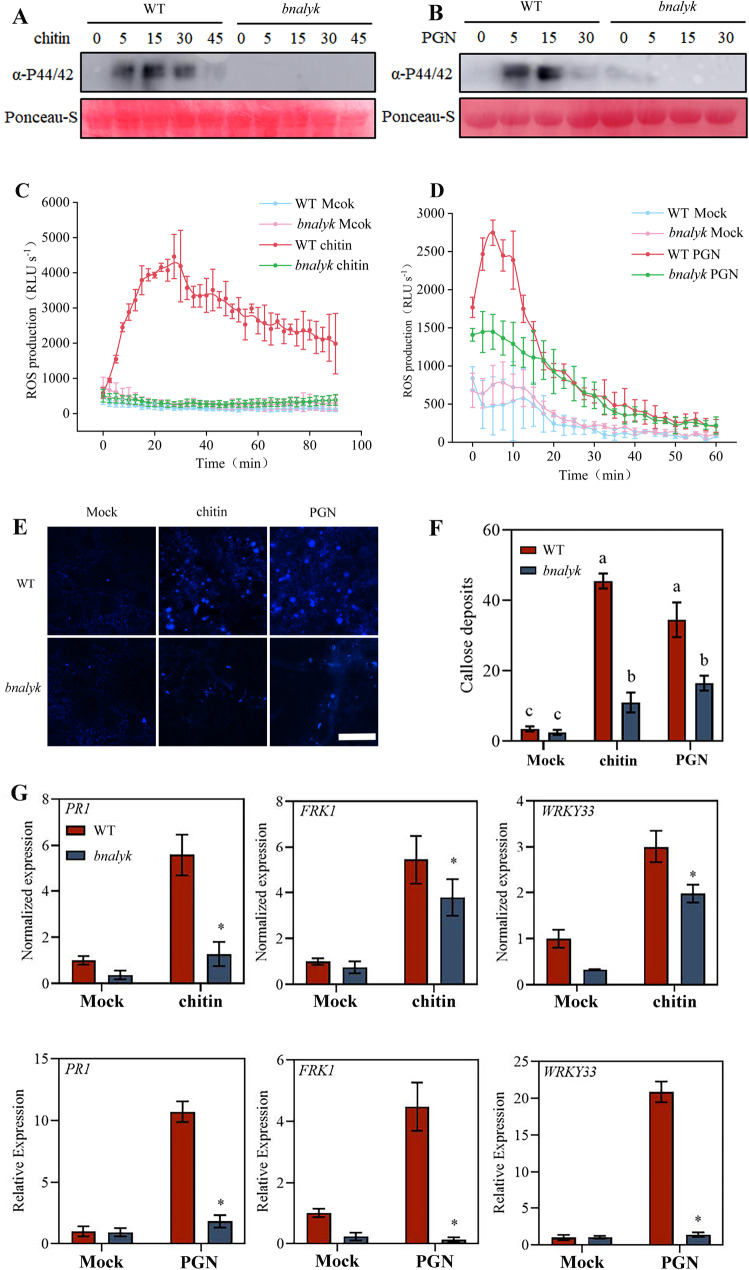
The *bnalyk* mutants exhibit compromised immune responses induced by chitin and PGN. Immunoblot analysis of MAPK phosphorylation (α-P44/42) in WT and *bnalyk* mutant plants after treatment with chitin (**A**) or PGN (**B**) over a time course. Production of reactive oxygen species (ROS) in response to chitin (**C**) or PGN (**D**) treatment. (**E**, **F**) Microscope images of callose deposition induced by chitin or PGN in WT or *bnalyk *mutants. (**G**) Expression analysis of *PR1*, *FRK1* and *WRKY33* genes induced by chitin or PGN in WT or *bnalyk* mutants. Student’s *t*-test: different letters indicate the values are significantly different, *P* < 0.05, *:* P* < 0.05.

Hallmark PAMP-triggered immunity (PTI) responses in which BnaLYK is involved during resistance to *S. sclerotiorum* were investigated. In WT plants, both chitin and PGN induced rapid ROS accumulation, peaking within minutes of treatment (Fig. 5C and D). In contrast, *bnalyk* mutants failed to mount ROS bursts following chitin treatment. Interestingly, PGN-pretreated *bnalyk* plants exhibited transiently elevated ROS levels during early induction (within 15–30 min), but sustained ROS production was significantly lower than in WT plants. Callose deposition assays demonstrated a striking genotype-dependent response. While WT plants showed robust callose accumulation following chitin or PGN treatment, *bnalyk* mutants displayed significantly reduced callose deposition (Fig. 5E, F). Compared to the Mock treatment, *bnalyk* mutants pretreated with chitin or PGN only exhibited marginal increases in callose levels, which remained far below WT levels. Gene expression profiling further highlighted the role of *BnaLYK* in defense signaling. Three hours post-PAMP treatment, *PR1* (pathogenesis-related protein 1), *FRK1* (flagellin-induced receptor-like kinase 1), and *WRKY33* (a downstream defense regulator) were significantly downregulated in *bnalyk* mutants compared to WT plants (Fig. 5G). Moreover, MAPK cascade was not detected in the *bnalyk* mutants after chitin or PGN treatment (Figs. 5A and B). The mutation disrupts the plant’s ability to fully activate defense signaling pathways downstream of chitin or PGN perception, thereby impairing immune responses against fungal pathogens like *S. sclerotiorum*. This underscores *BnaLYK* as a key regulator linking PAMP recognition to downstream defense activation in *B. napus*.

## Discussion

This study demonstrates that BnaLYK, specifically the homologous genes BnaA05.LYK and BnaC05.LYK, functions as immune receptors in *B. napus* governing resistance to necrotrophic fungus *S. sclerotiorum*. Our findings establish that BnaLYK is essential for chitin-triggered immunity, modulating hallmark PTI response including ROS burst, callose deposition, and defense gene activation, thereby contributing to basal resistance against SSR.

The identification of BnaA05.LYK and BnaC05.LYK as functional orthologs of Arabidopsis AtLYK1 is consistent with the conserved role of LYKs in fungal chitin perception across plant species [32]. Their plasma membrane localization, conserved protein architecture featuring extracellular LysM domains and intracellular kinase domains, and their ability to fully complement the chitin-insensitive phenotype of the Arabidopsis *atlyk1* mutant collectively support their role as genuine functional equivalents in the rapeseed immune system [33]. The induction of both *BnaLYK* genes following *S. sclerotiorum* infection further suggests their involvement in the defense response against this pathogen in plants.

*B. napus* is an allotetraploid species with substantial gene redundancy, which not only hinders the identification of resistance genes against *S. sclerotiorum*, but also poses challenges for constructing mutants using the CRISPR/Cas9 system [34]. Our findings demonstrate that *BnaA05.LYK* and *BnaC05.LYK* are functionally conserved in recognizing PAMPs and activating downstream immune pathways. The upregulation of these genes following *S. sclerotiorum* inoculation and their ability to restore chitin-induced immune responses in *Atlyk1* mutant, underscore their significance in PTI. Notably, CRISPR/Cas9-mediated knockout of *BnaLYK* compromised rapeseed resistance to *S. sclerotiorum*. This finding aligns with previous studies in *Arabidopsis*, where *Atlyk1* mutations increased susceptibility to fungal pathogens, reinforcing the evolutionary conservation of LysM receptor kinases in plant immunity [35].

A central finding of this work is that CRISPR/Cas9-mediated knockout of BnaLYK significantly compromises rapeseed resistance to *S. sclerotiorum*, as evidenced by enlarged necrotic lesions and suppressed expression of defense-related genes *FRK1*, *PR1*, *WRKY33*, and *PAD3*. FRK1, a marker of innate immunity, is known to be regulated by MAPK signaling in response to patterns [36]. Several of the PR proteins have been shown to have direct antimicrobial and antifungal properties [37]. WRKY33 is a key transcription factor that directly activates *PAD3* and other defense genes during responses to *S. sclerotiorum* [38]. MAPK cascade also mediated phosphorylates the substrate *WRKY33* [31]. There is currently no evidence indicating that WRKY33 is involved in the regulation of *PR1* expression. However, LYK1 was identified to phosphorylate the downstream receptor-like cytoplasmic kinase AVRPPHB SUSCEPTIBLE 1-LIKE 27 (PBL27), which in turn triggers the activation of a MAPK cascade [39]. The MAPK cascade leads to the expression of *PR1* and *FRK1*. This provides direct genetic evidence that BnaLYK is a positive regulator of SSR resistance. Importantly, our results delineate the specific role of BnaLYK in PAMP-triggered immunity. The failure of chitin pretreatment to enhance resistance in the BnaLYK mutants, in stark contrast to the strong induced resistance observed in wild-type plants, unequivocally demonstrates that BnaLYK is indispensable for chitin-induced resistance against SSR. This aligns with the established function of LYKs as core components of the chitin perception machinery.

The analysis of downstream immune outputs revealed a nuanced signaling cascade. The ablation of chitin-induced ROS burst and callose deposition in the mutants confirms that BnaLYK is required for these early PTI responses [40]. The differential response to PGN was particularly intriguing; while the ROS burst was initiated but not sustained, callose deposition was severely attenuated. LYKs in Arabidopsis are known to bind chitin, their direct binding to bacterial PGN remains controversial and is typically mediated by peptidoglycan recognition proteins (PGRPs, PGLYRPs) like AtLYM1/AtLYM3 [41]. AtLYK1 which does not bind to PGN but is required for signal transduction [42]. PGN receptors have also been identified in rice, where the LysM proteins OsLYP4 and OsLYP6, along with OsLYK1, are essential for the perception of both PGN and chitin [43, 44]. This suggests that BnaLYK may play a partially overlapping or cooperative role in the perception of certain bacterial PAMPs, potentially through interaction with other receptors, but is absolutely critical for mounting a full and effective defense response. The downregulation of key defense genes in mutant backgrounds upon PAMP treatment further underscores BnaLYK’s role in orchestrating the transcriptional reprogramming necessary for immunity. The fact that MAPK activation remained unchanged suggests that BnaLYK operates either upstream of MAPK cascades or within a parallel signaling branch that converges later to activate specific defense outputs [45].

Functional redundancy between homoeologs is widespread in *B. napus*. For example, mutations in both homologous genes of *BnaZEP* (*BnaA09.ZEP* and *BnaC09.ZEP*) were required to affect carotenoid biosynthesis [46]. WRKY28 responded to *S. sclerotiorum* and hormone treatments in *B. napus*. Five copies of *BnaWRKY28* were identified on chromosomes A01, C01, A03, A08, and C08 [31]. The functional redundancy is often inherent in allopolyploid genomes like that of *B. napus* (AACC) can complicate genetic studies. Here, we show that simultaneous mutation of both homoeologs, BnaA05.LYK and BnaC05.LYK, was necessary to achieve a strong loss-of-function phenotype, highlighting the potential functional redundancy between subgenomes. Overcoming this redundancy was key to elucidating the non-redundant essential role of the LYK pathway in SSR defense.

In conclusion, our study identifies BnaLYK as a master regulator of chitin-triggered immunity and a major determinant of resistance to *S. sclerotiorum* in rapeseed. It bridges the gap between model system discoveries in Arabidopsis and the practical needs of a major crop, providing a mechanistic understanding of PTI in *B. napus*. These findings offer a robust theoretical foundation for understanding plant-fungal interactions in polyploids and present a compelling practical target for molecular breeding. Engineering rapeseed cultivars with enhanced or modulated BnaLYK expression represents a promising and sustainable strategy for improving durable resistance to the devastating SSR. Future studies should focus on elucidating genetic redundancy within the LYK family and exploring potential synergies between BnaLYK and other immune receptors to further clarify functional similarities and differentiation.

## Supplementary Information

Supplementary Material 1.

Supplementary Material 2.

## Data Availability

The datasets used and/or analyzed during the current study are available from the corresponding author upon reasonable request.
