# Supplementary material for: CRISPR/Cas9-based knockout of BnaLYK compromises pattern-triggered immunity and resistance to Sclerotinia sclerotiorum in Brassica napus
Source: BMC Plant Biol. 2026 Apr 13;26:665. doi: 10.1186/s12870-025-07823-w (PMC13072592; doi:10.1186/s12870-025-07823-w)
Supplement: Supplementary file 2 — Supplementary Material 2. [file 12870_2025_7823_MOESM2_ESM.pdf]

Unprocessed figures

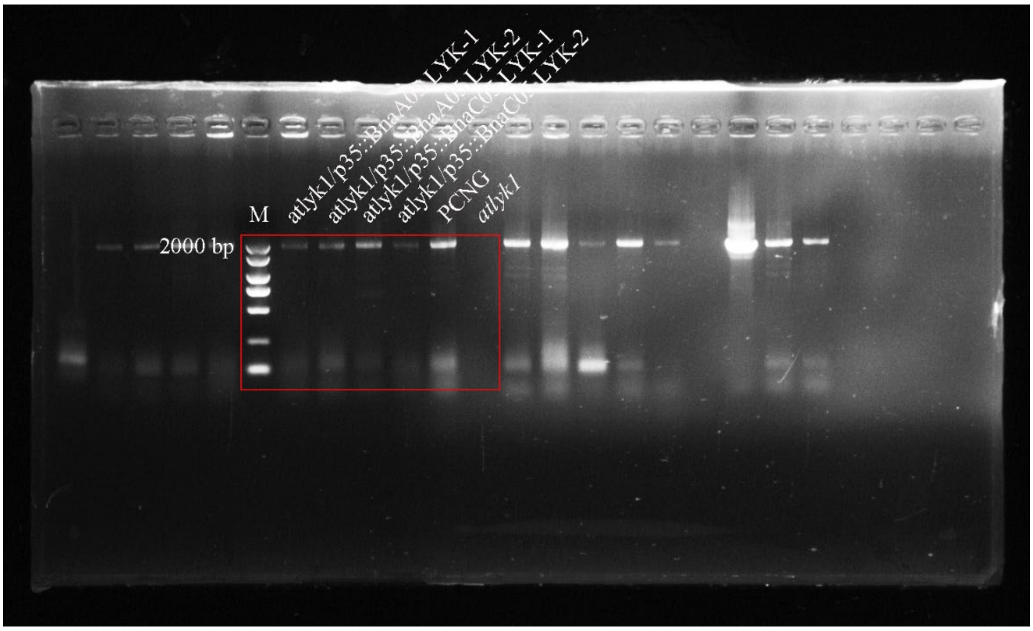

Fig. 2A. Screening for *atlyk1*/p35S::BnaLYK plants.

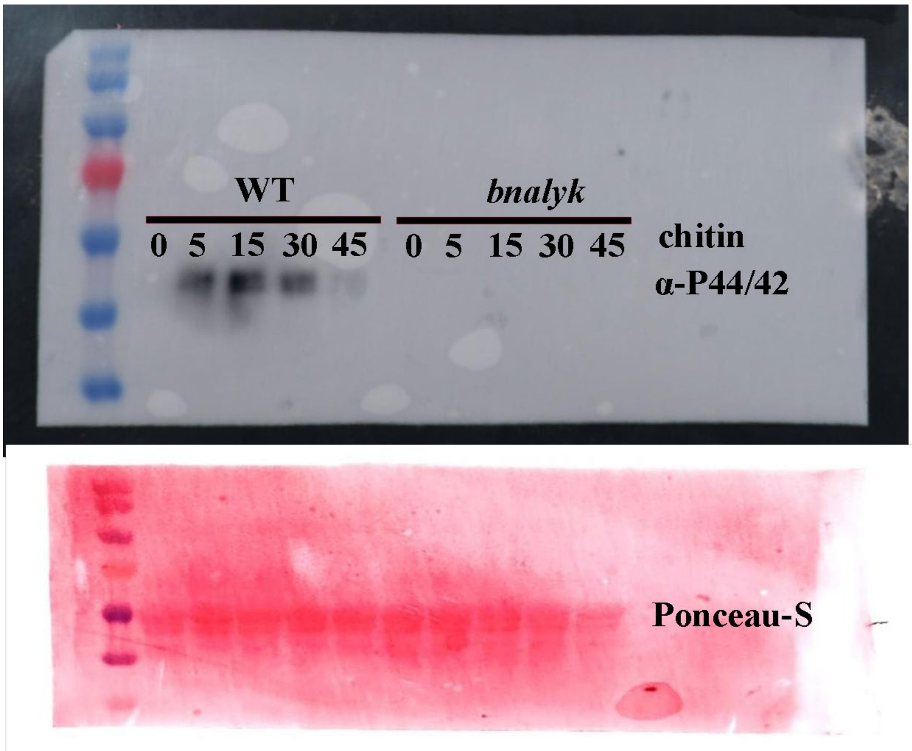

Fig. 5A. Immunoblot analysis of MAPK phosphorylation (α-P44/42) in WT and *bnalyk* mutant plants after treatment with chitin over a time course.

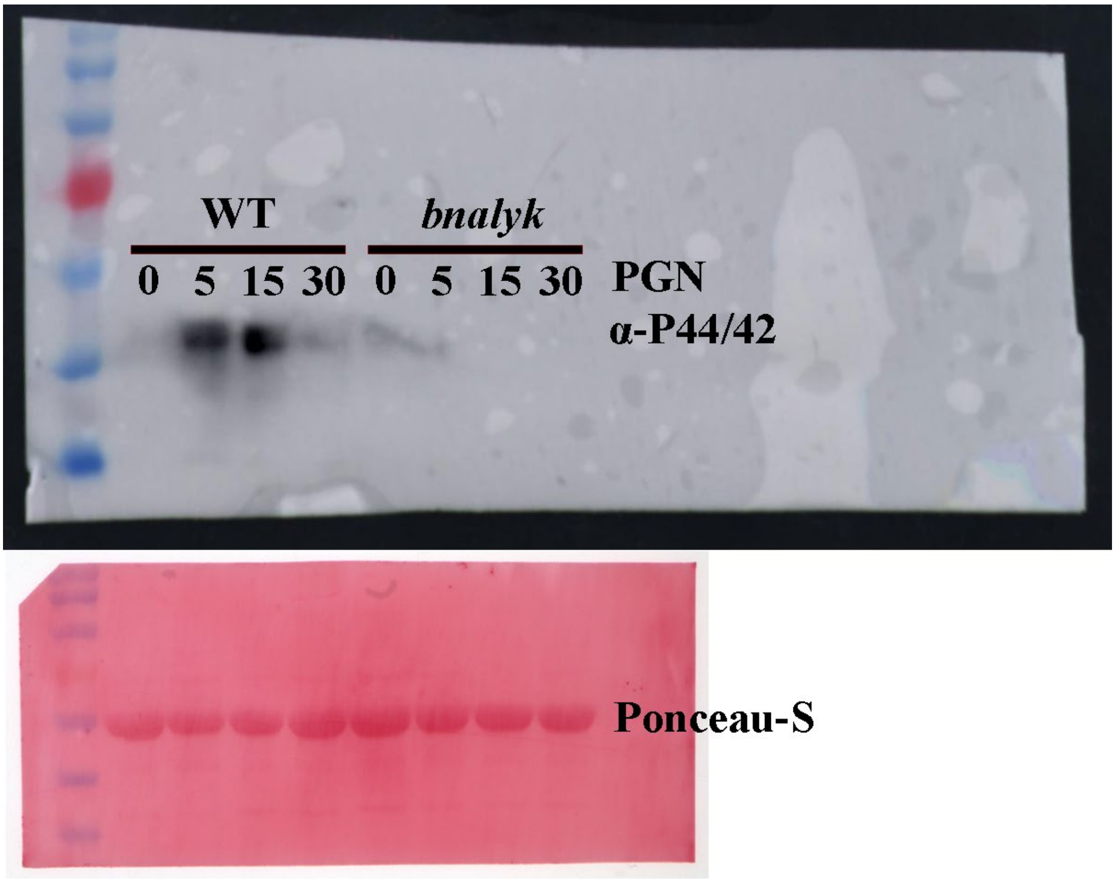

Fig. 5B. Immunoblot analysis of MAPK phosphorylation (α-P44/42) in WT and *bnalyk* mutant plants after treatment with peptidoglycan (PGN) over a time course.
